# Impact and Strategies on Joint Surgery Centers without Lockdowns during the Peak of the COVID-19 Pandemic: A Multicenter Cross-Sectional Study

**DOI:** 10.3390/jcm10225392

**Published:** 2021-11-19

**Authors:** Chia-Hao Hsu, Nin-Chieh Hsu

**Affiliations:** 1 Department of Orthopedics, Kaohsiung Municipal Ta-Tung Hospital, Kaohsiung 80145, Taiwan; ecowarrior.tw@yahoo.com.tw; 2 Department of Orthopedics, Kaohsiung Medical University Hospital, Kaohsiung 80756, Taiwan; 3 Department of Orthopedics, College of Medicine, Kaohsiung Medical University, Kaohsiung 80708, Taiwan; 4 Department of Internal Medicine, National Taiwan University Hospital, Taipei 10002, Taiwan; 5 Department of Internal Medicine, Zhongxing Branch, Taipei City Hospital, Taipei 10341, Taiwan

**Keywords:** COVID-19, pandemic, psychological effect, fear, lockdown, restriction, surgery scheduling strategies, joint surgery

## Abstract

The real psychological impact of COVID-19 remains difficult to quantify and may differ between hospital sizes and levels. Taiwan’s response to COVID-19 differed in that it successfully prevented its spread, without implementing any lockdowns before May 2021. Patients’ fear would be the major reason for the reduction of surgeries. The daily data for patients who underwent total knee arthroplasty (TKA), total hip arthroplasty, and hemiarthroplasty were collected from two major joint surgery centers of a university hospital and a community hospital in Taiwan. Compared with the previous year, the initial impact of the pandemic evidently reduced the total number of patients (outpatient: 20–29%; admission: 22–37%; surgery: 18–35%) in both hospitals. During the study period, the total number of TKAs decreased by 56–61% in both hospitals. The reduction in arthroplasty surgeries was attributable to patients’ fear. Even with confirmed COVID-19 cases, the university hospital experienced less impact than the community hospital. The TKA was the most affected of all surgery types. Even without local epidemics and restrictions in Taiwan, the worldwide pandemic inevitably led to a reduction of approximately 60% of the total TKA operation volume, especially for community hospitals. The surgery scheduling strategies helped maintain the routine arthroplasty services.

## 1. Introduction

The case of infection with novel coronavirus disease (COVID-19) was first reported in Wuhan, Hubei Province, China in December 2019. It then spread further and rapidly around the world. On 12 March 2020, The World Health Organization declared COVID-19 a global pandemic [1]. To date, over 220 million cases have been confirmed, and more than 4.5 million people have died from this virus [2]. The COVID-19 pandemic has significantly influenced the global economy and the healthcare system.

Based on our previous experience of the SARS outbreak in 2003, the Taiwanese government responded quickly to contain the COVID-19 outbreak [3,4]. The Taiwanese government has adopted an epidemic prevention strategy and requests people to put on a mask in public, to avoid gathering in public places, and to monitor self-health. Home quarantine for 14 days is required for all inbound travelers. For hospitals, body temperature is measured. Further, recent travel and exposure history are requested before people visit hospitals. As of 14 December 2020, Taiwan recorded more than 200 consecutive days with no new local cases. A total of 740 cases were confirmed and only seven people died from this virus in Taiwan. This indicates that Taiwan has managed and contained the further spread of COVID-19 [5].

Although the first wave of the COVID-19 outbreak has been successfully prevented in Taiwan, the comprehensive epidemic prevention strategy enacted by the Taiwanese government significantly influences the healthcare system [6,7]. Even without any shutdown or overburdening of medical services, a decreased rate of outpatients and surgeries for orthopedic problems was still observed in Taiwan. Patients’ fear of the pandemic was assumed to be the major reason behind the reduced surgeries. Many other countries have experienced the suspension of medical services. Although orthopedic doctors will not be the frontline staff to fight for the pandemic, there are still many orthopedic patients who need surgery during this period.

Several strategies have been implemented in Taiwan to prevent the first wave of COVID-19 outbreaks [8]. However, since the epidemic situation is not grave in Taiwan, there has been no need for restrictions or for changes in medical administrative measures. Compared with several other countries that have suffered complete or partial shutdown in medical services [9,10,11,12,13,14,15,16,17,18,19,20,21,22,23,24,25,26,27,28,29,30,31,32,33,34,35] and have had an impact on arthroplasty surgery [36,37,38,39,40], Taiwan does not have any policies that try to restrict the number of outpatients and limit the arrangements for routine elective joint replacement surgeries. Therefore, most of the reduction in the volume of medical services could be attributed to COVID-19-related psychological effects, such as the patients’ fear, rather than the medical institution itself.

**Figure 1:**
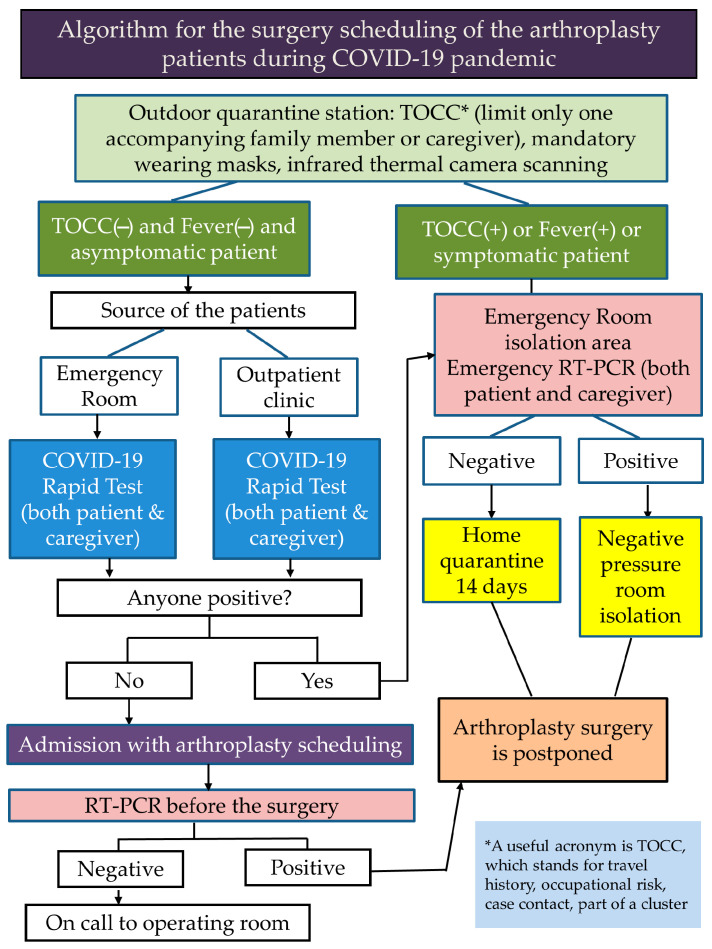
Strategies for scheduling arthroplasty surgery implemented at university and community hospitals in Taiwan during the COVID-19 pandemic. Considering that Taiwan has successfully controlled community or local transmission, the triage has clearly reduced patients’ fear and maintained the routine arthroplasty services. At the beginning of the pandemic, due to limited capacity and speed of the COVID-19 Rapid Test and RT-PCR, these strategies were used only for scheduling all elective surgeries, including arthroplasty surgeries. As the capacity and speed of the COVID-19 Rapid Test and RT-PCR gradually increased, all other types of surgery could also be applied.

In other countries, some hospitals have developed a process for scheduling patients for admission to reduce the risk of cross-infection of COVID-19 in hospitals during the pandemic [41,42]. Our hospital correspondingly developed a special process to schedule arthroplasty patients (Figure 1).

Fortunately, we have not encountered any cases where patients who tested negative turned positive after the surgery at the ward; although, it may happen. We strictly ensure that patients and their family members or caregivers only enter the ward after being tested negative through an RT PCR test. Not to mention, since the false negative rate of RT PCR is far lower than that of a rapid test, no negative reports have turned positive so far. In a case where a negative test does turn positive, the regulations of the hospital ensure that all contact points of the patients, their family members, and their caregivers are urgently transferred to the negative pressure isolation room, and the ward is temporarily closed for cleaning and disinfection.

Although this process may psychologically affect the patients’ willingness to visit the hospital, considering the initial success in blocking the first wave transmission, Taiwan’s strategies may help maintain routine elective surgery by reducing patients’ fear of infection during surgical procedure. With hospital checks and specific infection control strategies, such as traffic control bundling [4,43,44,45], most patients feel safe and confident when they enter hospitals. However, it is still unavoidable that some patients will not go to the hospitals at all due to fear. In the face of possible local or community contagion, hospitals are highly vigilant, and will not ignore asymptomatic cases [46,47].

In previous impact analyses of all types of orthopedic surgeries, total knee arthroplasty (TKA) seemed to be the most severely affected type of surgery [48]. Our institutions are the major hospitals that perform TKA and total hip arthroplasty (THA) surgeries in southern Taiwan. However, the actual impact of the COVID-19 pandemic on TKA, THA, and hemiarthroplasty surgeries at different levels of hospitals in Taiwan is still unclear and needs to be further clarified. This study aimed to investigate the changes in hip and knee arthroplasty surgeries at one university hospital (medical center) and one community hospital in Taiwan during the initial peak period of the COVID-19 pandemic, compared with the previous year. Our results could help us understand the pure psychological impact of the COVID-19 pandemic on hip and knee arthroplasty surgeries in Taiwan.

To our knowledge, this is the first study quantifying the initial impact of the COVID-19 pandemic on arthroplasty surgery conducted in different levels of hospitals in a country without any established restrictions or lockdowns. This study has is unique as it focuses on the least infected area, while other studies have targeted severely infected areas. Thus, it effectively demonstrates the impact of pure psychological factors, which are not directly influenced by the pandemic. Taiwan had only a few local cases during the first wave. Thus, it could be the optimal place for our study.

## 2. Materials and Methods

### 2.1. Data Acquisition and Study Design

This study was approved by the Institutional Review Board (IRB) of Kaohsiung Medical University Hospital (KMUH) with an approval number: KMUHIRB-EXEMPT (II)-2020. This study used retrospective comparison analysis. The study period ran from 1 March 2020 to 25 April 2020, for a total of 8 weeks. This period represented the peak of the initial impact of the pandemic. We compared the data obtained from the study period with a control group. The control group was a corresponding period of the last year (1 March 2019 to 25 April 2019). Overall information on orthopedic patients was obtained monthly from two hospitals’ administration office, including medical information about outpatient visits, inpatient hospital admissions, and surgical records. Names and other personal information had been removed from the data. We did not ask the patients to complete any questionnaire at the time of the study, which was already more than six months after the first wave of the pandemic. We believe that due to the time elapsed, there could be recall bias in the participants’ responses. Thus, the data collected did not include questionnaire responses. Instead, the hospital’s performance statistics obtained from its administrative department would be more accurate and precise, and would not change over time (i.e., these would be scientifically reproducible). Thus, we believe that these data would be better than that collected from questionnaires.

Surgical methods were classified as: total types of orthopedic surgery, TKA (including revisions), THA (including revisions), hemiarthroplasty, emergency surgery, and elective surgery. Due to the number of revision arthroplasty being very small, with no more than two operations per week, it was included in the primary arthroplasty. Each of these categories has its analytical characteristics, but there must be some overlaps. For example, TKA or THA is certainly one of the elective surgeries. Daily data for patients receiving TKA, THA, and hemiarthroplasty including the number, sex, and age of patients were collected from two different levels of hospitals: a university hospital/medical center (Kaohsiung Medical University Hospital), and a community hospital (Kaohsiung Municipal Ta-Tung Hospital). These two hospitals were the major joint reconstructive surgery centers in Kaohsiung city (population 2.77 million), the largest city in southern Taiwan and the second largest metropolitan in Taiwan.

### 2.2. Statistical Analysis

All the statistical analyses were processed using the statistical software package SPSS (IBM Corp., Armonk, NY, USA). Descriptive statistics were calculated to determine the major characteristics of the patients. Categorical data were presented as frequencies, and continuous data were presented as mean ± standard deviation (SD). A two-sample t-test was used to compare continuous data between the two periods. The Chi-square test was used to compare categorical data between the two periods. For all the analyses, a *p*-value < 0.05 was considered a significant difference.

## 3. Results

### 3.1. Overall Impact

#### 3.1.1. The Community Hospital

In total, 17,039 outpatients were analyzed. There were 7308 visits (43%) in March and April 2020, and 9731 visits (57%) in March and April 2019. The amount of outpatient visits reduced by 29% in March and 20% in April during the study period, respectively, as compared with that during the previous year. In total, 2541 hospital admissions were analyzed. There were 1032 admissions (41%) that occurred in March and April 2020, and 1509 admissions (60%) in March and April 2019. The number of inpatients shrank by 27% in March and 37% in April of 2020, respectively, as compared with that in 2019. In total, 619 surgical patients who underwent orthopedic surgery were recorded. There were 261 patients (42%) undergoing orthopedic surgery in 2020 and 358 patients (58%) in 2019. The number of patients undergoing orthopedic surgery shrank by 35% in March and 18% in April of 2020, respectively, compared with that in 2019.

#### 3.1.2. The University Hospital

In total, 22,800 outpatients were included. There were 9872 visits (43%) in March and April 2020, and 12,928 visits (57%) in March and April 2019. The amount of outpatient visits reduced by 22% in March and 26% in April during the study period, respectively, compared with that in 2019. In total, 5780 hospital admissions were analyzed. There were 2417 admissions (42%) that occurred in March and April 2020, and 3363 admissions (58%) in March and April 2019. The number of inpatients shrank by 22% in March and 35% in April of 2020, respectively, compared with that in 2019. In total, 1432 surgical patients who underwent orthopedic surgery were recorded. There were 591 patients (41%) undergoing orthopedic surgery in 2020 and 841 patients (59%) in 2019. The number of patients undergoing orthopedic surgery shrank by 26% in March and 34% in April of 2020, respectively, compared with that in 2019.

### 3.2. TKA

#### 3.2.1. The Community Hospital

**Figure 2:**
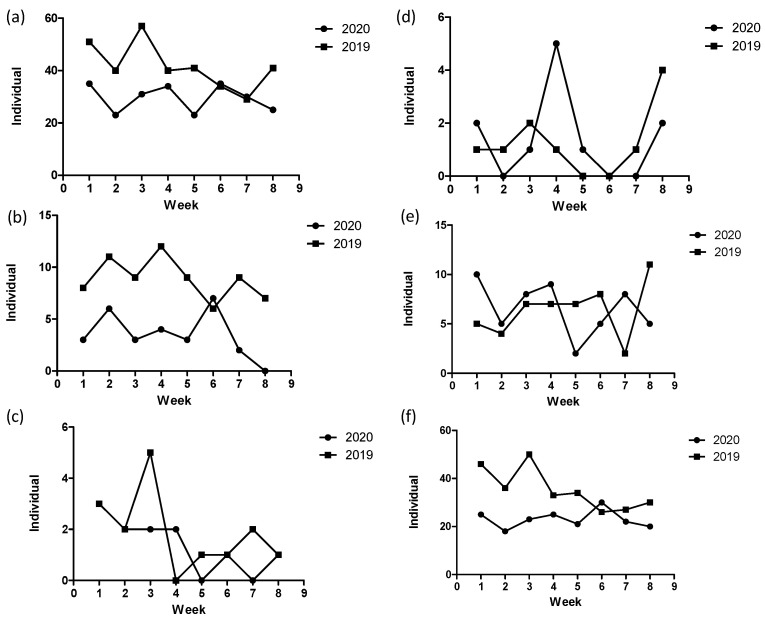
Weekly number of patients receiving the (**a**) total types of surgery, (**b**) total knee arthroplasty, (**c**) total hip arthroplasty, (**d**) hemiarthroplasty, (**e**) emergency surgery, and (**f**) elective surgery at the community hospital during the study (2020) and control (2019) periods.

**Figure 3:**
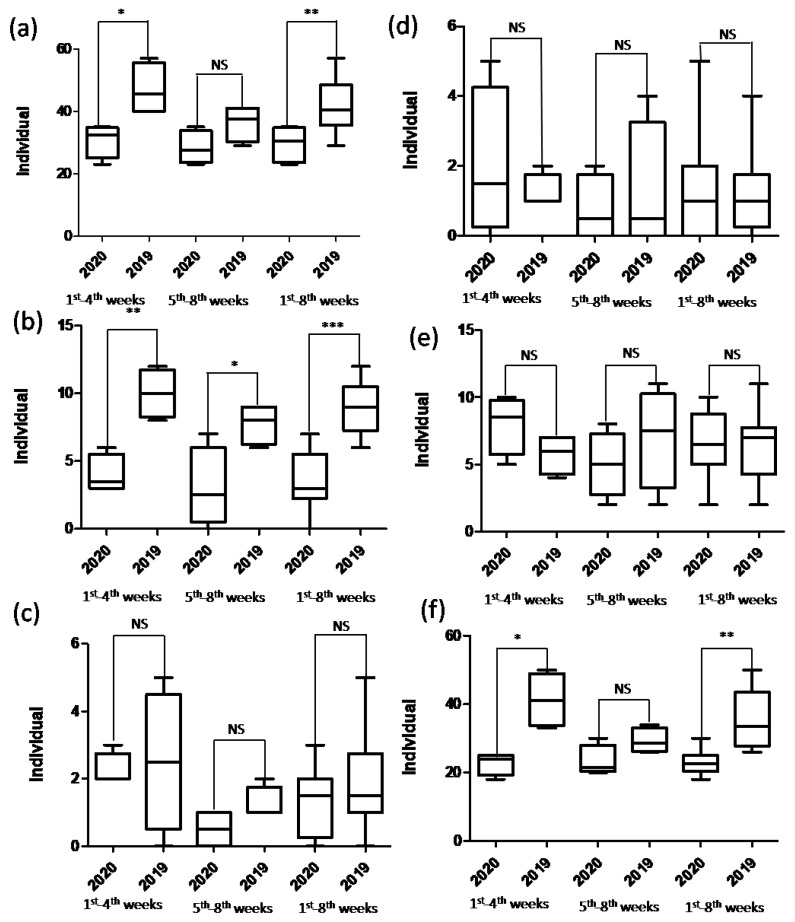
Box plots of the number of patients receiving the (**a**) total types of surgery, (**b**) total knee arthroplasty, (**c**) total hip arthroplasty, (**d**) hemiarthroplasty, (**e**) emergency surgery, and (**f**) elective surgery at the community hospital during the study (2020) and control (2019) periods. * indicates *p* < 0.05. ** indicates *p* < 0.01. *** indicates *p* < 0.001. “NS” indicates insignificant.

The total number of TKAs indicated a patient decrease from 71 in the control period to 28 (61% reduction) in the year of study. The mean age of patients in the study period (70.2 ± 6.6 years) was significantly younger (*p* = 0.044) than that in the control period (73.1 ± 6.4 years) for the full period (Table 1). No significant difference was found in the mean age between the two periods for the first and last 4 weeks. Sex ratios between the two periods differed insignificantly for the first and last 4 weeks and the full period (Table 1). The weekly number of TKAs in the study period was less than that in the control period, except for the 6th week (Figure 2b). The mean number of TKAs in the study periods was significantly less than that in 2019 for the first 4 weeks (*p* < 0.01), last 4 weeks (*p* < 0.05), and the full period (*p* < 0.001) (Figure 3b).

#### 3.2.2. The University Hospital

**Figure 4:**
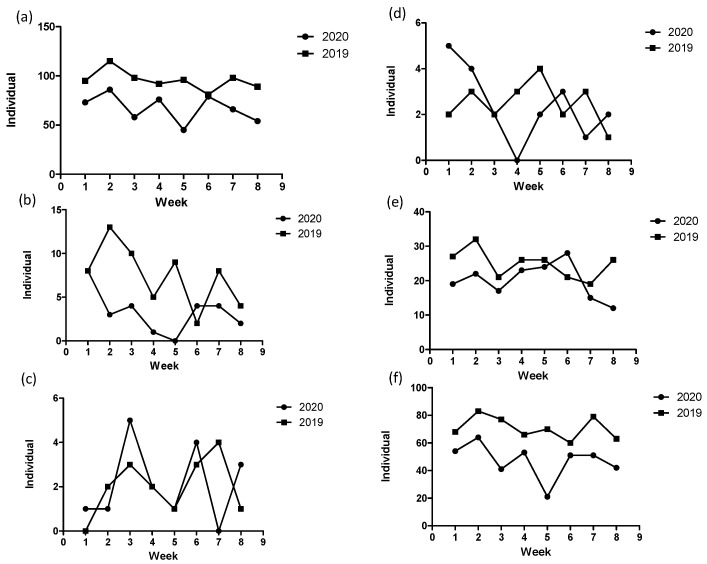
Weekly number of patients receiving the (**a**) total types of surgery, (**b**) total knee arthroplasty, (**c**) total hip arthroplasty, (**d**) hemiarthroplasty, (**e**) emergency surgery, and (**f**) elective surgery at the university hospital during the study (2020) and control (2019) periods.

**Figure 5:**
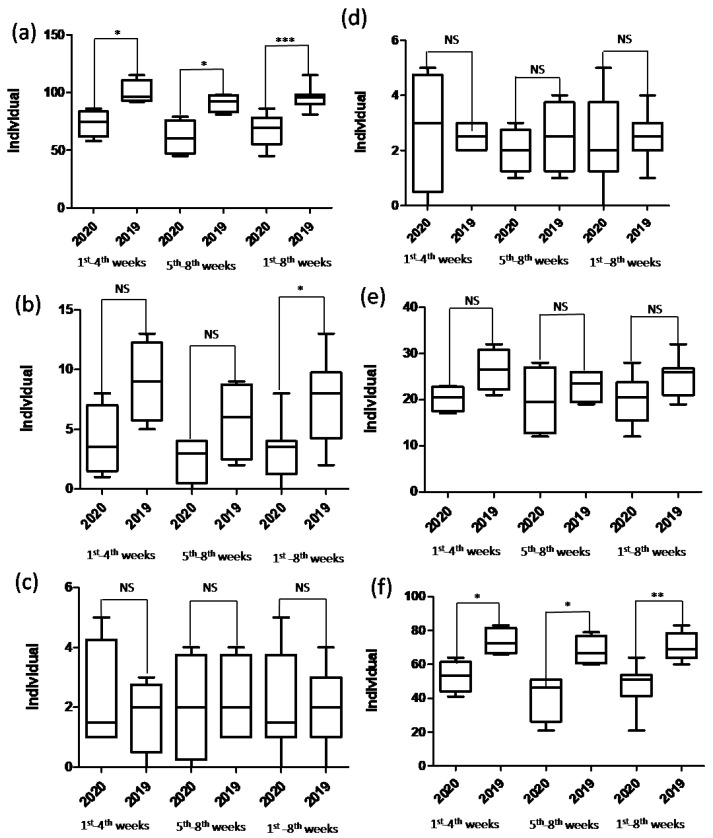
Box plots of the number of patients receiving (**a**) total types of surgery, (**b**) total knee arthroplasty, (**c**) total hip arthroplasty, (**d**) hemiarthroplasty, (**e**) emergency surgery, and (**f**) elective surgery at the university hospital during the study (2020) and control (2019) periods. * indicates *p* < 0.05. ** indicates *p* < 0.01. *** indicates *p* < 0.001. “NS” indicates insignificant.

The total number of TKAs indicated a patient decrease, that is, from 59 in the control period to 26 (56% reduction) in the study period. There were no significant differences between the two periods for the full period and the first and last 4 weeks regarding the mean age and sex ratio (Table 2). The weekly number of TKAs in the study period was less than that in the control period, except for the 1st and 6th weeks (Figure 4b). The mean number of TKAs in the study period was significantly less than that in 2019 for the full period (*p* < 0.05). However, no significant differences were observed for the first and last 4 weeks (Figure 5b).

### 3.3. THA

#### 3.3.1. The Community Hospital

The total number of THAs indicated a patient decrease, that is, from 15 in the control period to 11 (27% reduction) in the study period. There were no significant differences between the two periods in the mean age and sex ratio for the first and last 4 weeks and the full period (Table 1). There was no consistent trend of changes in the weekly number of THAs between the two periods (Figure 2c). The mean number of patients in the first and last 4 weeks and the full period (Figure 3c) differed insignificantly.

#### 3.3.2. The University Hospital

The total amount of THAs (17 patients) in the study period was similar to that (16 patients) in the control period. The mean age and sex ratio between the two periods differed insignificantly, except for the sex ratio (F/M = 14/3 for the study period and F/M = 8/8 for the control period, *p* = 0.049) for the full period (Table 2). There was no consistent trend of changes in the weekly number of THAs between the two periods (Figure 4c). The mean number of patients for the first and last 4 weeks and the full period differed insignificantly (Figure 5c).

### 3.4. Hemiarthroplasty

#### 3.4.1. The Community Hospital

The total number of patients receiving hemiarthroplasty surgery (11 patients) in the study period was similar to that (10 patients) in the control period. The mean age and sex ratio differed insignificantly between the two periods (Table 1). There was no consistent trend of changes in the weekly number of hemiarthroplasties between the study and control periods (Figure 2d). The mean number of patients for the first and last 4 weeks and the full period differed insignificantly (Figure 3d).

#### 3.4.2. The University Hospital

The total number of patients that received hemiarthroplasty surgery (19 patients) in the study period was similar to that (20 patients) in the control period. The mean age and sex ratio between the two periods differed insignificantly (Table 2). There was no consistent trend of changes in the weekly number of hemiarthroplasties between the two periods (Figure 4d). The mean number of TKAs for the first and last 4 weeks and the full period differed insignificantly (Figure 5d).

## 4. Discussion

This is the first study quantifying the initial impact of the COVID-19 pandemic on hip and knee arthroplasty conducted in different levels of hospitals in a country without any established restrictions. Generally, the results showed that the total numbers of outpatients, hospital admissions, and orthopedic surgeries in the period of the pandemic were patently less than the numbers in the previous year in March and April at the two hospitals. The two hospitals recorded a significant shrinkage in the average weekly number of patients undergoing elective surgery and all types of orthopedic surgery during the COVID-19 pandemic period in March. The university hospital experienced significantly less reduction on all types of orthopedic surgery than the community hospital in March.

For the entire period, the mean age of TKA patients was significantly younger during the COVID-19 pandemic period compared with the control period in the community hospital. However, this was not observed at the university hospital. The THA between the two periods regarding the age and number of the patients for the two hospitals differed insignificantly. The sex ratio for THA at the university hospital for the full period differed significantly. In other words, significantly more women received THA during the pandemic. However, the sample size of THAs was small, so we think this may be just a coincidence.

We found that the total number of TKAs had patently decreased by 56% and 61% for both hospitals during the COVID-19 pandemic period. Although Taiwan had successfully contained the COVID-19 outbreak, its impact seemed to reduce the patients’ willingness to receive TKA during this period. These patients were elderly. Consequently, a high rate of mortality and complications had been reported among patients receiving surgical procedures during the incubation period for this virus [49]. Moreover, these patients did not need to undergo urgent elective TKA surgery. The surgeons may ask these patients to consider postponing their surgeries during this period. This situation in the THA was unclear as the number of patients receiving THA was relatively small.

The community and the university hospital were at two different levels. The community hospital, unlike the university hospital, did not provide any standard negative pressure isolation rooms for the confirmed COVID-19 patients. During the study period, some patients diagnosed with COVID-19 had been admitted in negative pressure rooms in our university hospital. We expected that the patients who needed to receive TKA or THA surgery may fear contracting this virus at our university hospital, causing more patients to cancel their appointments during this period. Surprisingly, this situation seemed not to be observed in the university hospital, compared with the community hospital. This is an unexpected and interesting result, and we can only speculate that patients might have been more confident in the university hospital, which was able to do well in infection control and provide better personal protective equipment.

We observed that the mean age of patients undergoing TKAs was significantly younger in the COVID-19 pandemic period than that in the control period in the community hospital. This indicated that elderly patients may fear COVID-19 more and tended to cancel or postpone the TKA surgery during this pandemic. This result seemed reasonable as elderly patients were more vulnerable and were not easy to recover from this virus. The news media broadcasting that the elderly may have a higher mortality rate if infected may additionally cause psychological stress. However, this situation was not observed at the university hospital.

We speculated that there were some possible reasons for this COVID-19-related psychological fear. First, public panic may have stemmed from media misinformation or the exaggeration of rumors. Second, to some extent, the panic could have been due to concerns about the lack of personal protective equipment, such as masks or other medical resources. Third, some hospitals inevitably had to admit some confirmed COVID-19 patients, which would cause a lot of psychological pressure on other inpatients in the same hospital, and people would be worried about whether they would be infected after admission. As we observed, patients may have been afraid to seek timely treatment due to psychological factors. This delay might have negatively affected the treatment process of certain diseases and in turn, influenced the functional results.

This study had some limitations. First, the sample size of THAs was quite small, and the COVID-19 impact on THA seemed unobserved. The sex ratio difference of THAs between the study (significantly more female patients) and control periods might have been due to the limited sample size, or it might have been a coincidence. Second, we could not retrospectively retrieve or reproduce the reasons for people’s unwillingness to visit hospitals during the pandemic; further, we could not obtain information about whether the procedures were completely cancelled or just postponed at the patient’s request. Not interrogating the patients on these aspects using questionnaires may have led to overlooking some other factors. Therefore, any other factors that affected this remain unclear. Third, the detailed disease grading of each surgery was not included in our data; therefore, we could not ascertain whether there was any difference between the community hospital and the university hospital in terms of the spectrum of disease grading. Fourth, although this study presented a psychological impact, we had no further information to clarify which psychological factors were the key factors, or what the mechanisms of avoidance or fear were. We believed that understanding all these factors could help us formulate the best strategies to reduce the impact of a pandemic on hospitals; therefore, future studies could investigate the issues affecting people’s willingness.

## 5. Conclusions

The COVID-19 pandemic still inevitably had a significant impact on a country without lockdowns or restrictions, accompanied by a significant psychological impact, especially in the first wave. Taiwan could be one of the best places to analyze and quantify the pure psychological effects. In Taiwan, the TKA or elective surgery patients during the pandemic were significantly younger than the control group in the community hospital. The initial impact of the pandemic reduced older people’s desire to receive TKA or elective surgery. Although some confirmed COVID-19 patients had been admitted exclusively in the university hospital, the university hospital still experienced less impact by the COVID-19 pandemic than the community hospital. Further, elective surgery had significantly more impact than non-elective surgery. A drop in the number of hip and knee arthroplasty surgeries in non-restricted Taiwanese hospitals during the COVID-19 pandemic was attributable to patients’ fear. TKA was the most affected and the most reduced of all surgery types. Even without any established restrictions, the initial impact of the pandemic unavoidably led to a shrinkage of approximately 60% of the total TKA operation volume. The surgery scheduling strategies helped maintain the routine arthroplasty services.

## Figures and Tables

**Table 1:** Major characteristics of patients receiving total knee arthroplasty, total hip arthroplasty, or hemiarthroplasty surgery at the community hospital during the study (2020) and control (2019) periods; ** *p* ≤ 0.01, * *p* ≤ 0.05.

	2020	2019		2020	2019		2020	2019	
	1–4 Weeks		*p*-Value	5–8 Weeks		*p*-Value	1–8 Weeks		*p*-Value
	All types of surgery								
Age	59.5 ± 18.0	62.1 ± 17.2	0.200	56.9 ± 18.6	61.5 ± 18.4	0.048 *	58.3 ± 18.3	61.8 ± 17.7	0.019 *
Sex (F/M)	118/70	79/44	0.794	73/40	97/48	0.700	152/84	215/118	0.969
	Total knee arthroplasty								
Age	70.1 ± 7.1	72.5 ± 6.8	0.245	70.3 ± 6.2	74.0 ± 5.9	0.080	70.2 ± 6.6	73.1 ± 6.4	0.044 *
Sex (F/M)	13/3	32/8	0.915	9/3	26/5	0.503	22/6	58/13	0.723
	Total hip arthroplasty								
Age	61.9 ± 14.3	62.3 ± 7.9	0.938	56.5 ± 3.5	53.8 ± 12.0	0.778	60.9 ± 13.0	59.5 ± 9.9	0.751
Sex (F/M)	6/3	4/6	0.245	2/0	4/1	0.495	8/3	8/7	0.315
	Hemiarthroplasty								
Age	79.6 ± 8.6	77.0 ± 11.1	0.640	72.3 ± 12.4	78.2 ± 5.3	0.347	77.6 ± 9.7	77.6 ± 8.2	0.993
Sex (F/M)	5/3	4/1	0.506	2/1	1/4	0.187	7/4	5/5	0.528
	Emergency surgery								
Age	60.1 ± 17.5	56.7 ± 23.0	0.540	54.5 ± 22.5	57.6 ± 19.0	0.597	57.9 ± 19.6	57.2 ± 20.6	0.863
Sex (F/M)	17/15	15/8	0.370	13/7	16/12	0.583	30/22	31/20	0.750
	Elective surgery								
Age	59.2 ± 18.1	62.8 ± 16.2	0.107	57.5 ± 17.7	62.5 ± 18.2	0.047 *	58.3 ± 17.9	62.7 ± 17.0	0.009 **
Sex (F/M)	62/29	103/62	0.361	60/33	81/39	0.470	122/62	184/98	0.814

The bold type indicates a significant difference. Note that these categories of surgeries were not mutually exclusive.

**Table 2:** Major characteristics of patients receiving total knee arthroplasty, total hip arthroplasty, or hemiarthroplasty surgery at the university hospital during the study (2020) and control (2019) periods; * *p* ≤ 0.05.

	2020	2019		2020	2019		2020	2019	
	1–4 Weeks		*p*-Value	5–8 Weeks		*p*-Value	1–8 Weeks		*p*-Value
	All types of surgery								
Age	49.2 ± 21.3	53.0 ± 20.5	0.017 *	50.8 ± 20.6	52.2 ± 20.3	0.382	49.9 ± 21.0	52.6 ± 20.4	0.018 *
Sex (F/M)	152/141	229/170	0.245	127/117	164/200	0.091	279/258	393/370	0.695
	Total knee arthroplasty								
Age	70.1 ± 6.2	70.2 ± 8.8	0.968	73.4 ± 7.6	72.4 ± 7.4	0.734	71.4 ± 6.8	71.1 ± 8.3	0.872
Sex (F/M)	13/3	28/8	0.777	7/3	16/7	0.980	20/6	44/15	0.817
	Total hip arthroplasty								
Age	60.6 ± 20.3	53.3 ± 15.6	0.448	68.1 ± 9.6	61.2 ± 19.2	0.373	64.1 ± 16.2	57.8 ± 17.6	0.288
Sex (F/M)	7/2	3/4	0.152	7/1	5/4	0.149	14/3	8/8	0.049 *
	Hemiarthroplasty								
Age	77.3 ± 6.5	80.5 ± 11.6	0.457	77.4 ± 9.7	76.8 ± 9.0	0.898	77.8 ± 7.9	78.7 ± 10.3	0.786
Sex (F/M)	7/3	5/5	0.361	5/3	8/2	0.410	13/6	13/7	0.821
	Emergency surgery								
Age	48.5 ± 23.3	55.6 ± 21.6	0.034 *	48.6 ± 21.2	49.4 ± 23.8	0.811	48.5 ± 22.2	52.7 ± 22.8	0.084
Sex (F/M)	41/40	57/48	0.602	32/47	36/56	0.855	73/87	93/104	0.638
	Elective surgery								
Age	49.4 ± 20.6	52.1 ± 20.0	0.146	51.8 ± 20.3	53.2 ± 18.9	0.470	50.5 ± 20.5	52.6 ± 19.5	0.104
Sex (F/M)	111/101	172/122	0.17	95/70	128/144	0.033	206/171	300/266	0.621

The bold type indicates a significant difference. Note that these categories of surgeries were not mutually exclusive.

## Data Availability

The data presented in this study are available on request from the first author.

## References

[1] Cucinotta D., Vanelli M. (2020). WHO declares COVID-19 a pandemic. Acta Biol. Med..

[2] COVID-19 Dashboard by the Center for Systems Science and Engineering (CSSE) at Johns Hopkins University. https://arcg.is/0fHmTX.

[3] Wang C.J., Ng C.Y., Brook R.H. (2020). Response to COVID-19 in Taiwan big data analytics, new technology, and proactive testing. JAMA.

[4] Yen M.-Y., Schwartz J., Chen S.-Y., King C.-C., Yang G.-Y., Hsueh P.-R. (2020). Interrupting COVID-19 transmission by implementing enhanced traffic control bundling: Implications for global prevention and control efforts. J. Microbiol. Immunol. Infect..

[5] Taiwan Centers for Disease Control CECC Confirms 4 More Imported COVID-19 Cases. Cases Arrive in Taiwan from Indonesia and the Philippines. https://www.cdc.gov.tw/En/Bulletin/Detail/0hKFLWF095r8WTnDDLgWfg?typeid=158.

[6] Su V.Y.-F., Yen Y.-F., Yang K.-Y., Su W.-J., Chou K.-T., Chen Y.-M., Perng D.-W. (2020). Masks and medical care: Two keys to Taiwan’s success in preventing COVID-19 spread. Travel. Med. Infect. Dis..

[7] Hsu C.-H., Chen C.-H., Huang H.-T., Yang C.-J., Chen Y.-H. (2021). To safely reopen after a lockdown, masks are crucial: Lessons from Taiwan. Public Health.

[8] Lee I.-K., Wang C.-C., Lin M.-C., Kung C.-T., Lan K.-C., Lee C.-T. (2020). Effective strategies to prevent coronavirus disease-2019 (COVID-19) outbreak in hospital. J. Hosp. Infect..

[9] De Filippo O., D’Ascenzo F., Angelini F., Bocchino P.P., Conrotto F., Saglietto A., Secco G.G., Campo G., Gallone G., Gaido L. (2020). Reduced rate of hospital admissions for ACS during Covid-19 outbreak in Northern Italy. N. Eng. J. Med..

[10] Athey A.G., Cao L., Okazaki K., Zagra L., Castelli C.C., Kendoff D.O., Kerr J.M., Yates A.J., Stambough J.B., Sierra R.J. (2020). Survey of AAHKS International Members on the Impact of COVID-19 on hip and knee arthroplasty practices. J. Arthroplast..

[11] Bedard N.A., Elkins J.M., Brown T.S. (2020). Effect of COVID-19 on hip and knee arthroplasty surgical volume in the United States. J. Arthroplast..

[12] Brown T.S., Bedard N.A., Rojas E.O., Anthony C.A., Schwarzkopf R., Barnes C.L., Stambough J.B., Mears S.C., Edwards P.K., Nandi S. (2020). The effect of the COVID-19 pandemic on electively scheduled hip and knee arthroplasty patients in the United States. J. Arthroplast..

[13] Park C., Sugand K., Nathwani D., Bhattacharya R., Sarraf K.M. (2020). Impact of the COVID-19 pandemic on orthopedic trauma workload in a London level 1 trauma center: The “golden month”. Acta Orthop..

[14] Phillips M.R., Chang Y., Zura R.D., Mehta S., Giannoudis P.V., Nolte P.A., Bhandari M. (2020). Impact of COVID-19 on orthopaedic care: A call for nonoperative management. Ther. Adv. Musculoskelet. Dis..

[15] Wong J.S.H., Cheung K.M.C. (2020). Impact of COVID-19 on orthopaedic and trauma service: An epidemiological study. J. Bone Jt. Surg..

[16] Ranuccio F., Tarducci L., Familiari F., Mastroianni V., Giuzio E. (2020). Disruptive effect of COVID-19 on orthopaedic daily practice: A cross-sectional survey. J. Bone Jt. Surg..

[17] Liebensteiner M.C., Khosravi I., Hirschmann M.T., Heuberer P.R., Thaler M. (2020). Massive cutback in orthopaedic healthcare ser-vices due to the COVID-19 pandemic: An online survey of almost 1400 orthopaedic surgeons in Austria, Germany and Switzerland. Knee Surg. Sport Traumatol. Arthrosc..

[18] Thaler M., Khosravi I., Hirschmann M.T., Kort N.P., Zagra L., Epinette J.A., Liebensteiner M.C. (2020). Disruption of joint arthroplasty services in Europe during the COVID-19 pandemic: An online survey within the European Hip Society (EHS) and the European Knee Associates (EKA). Knee Surg. Sport Traumatol. Arthrosc..

[19] Vermeşan D., Todor A., Andrei D., Niculescu M., Tudorache E., Haragus H. (2021). Effect of COVID-19 Pandemic on Orthopedic Surgery in Three Centers from Romania. Int. J. Environ. Res. Public Health.

[20] Magro F., Perazzo P., Bottinelli E., Possenti F., Banfi G. (2020). Managing a Tertiary Orthopedic Hospital during the COVID-19 Epidemic, Main Challenges and Solutions Adopted. Int. J. Environ. Res. Public Health.

[21] Tarantino U., Cariati I., Tancredi V., Casamassima D., Piccirilli E., Iundusi R., Gasbarra E. (2020). State of Fragility Fractures Management during the COVID-19 Pandemic. Int. J. Environ. Res. Public Health.

[22] Aprato A., Guindani N., Massè A., Castelli C.C., Cipolla A., Antognazza D., Benazzo F., Bove F., Casiraghi A., Catani F. (2021). Clinical Activities, Contaminations of Surgeons and Cooperation with Health Authorities in 14 Orthopedic Departments in North Italy during the Most Acute Phase of Covid-19 Pandemic. Int. J. Environ. Res. Public Health.

[23] Elran-Barak R., Mozeikov M. (2020). One Month into the Reinforcement of Social Distancing due to the COVID-19 Outbreak: Subjective Health, Health Behaviors, and Loneliness among People with Chronic Medical Conditions. Int. J. Environ. Res. Public Health.

[24] Ruzzini L., De Salvatore S., Lamberti D., Maglione P., Piergentili I., Crea F., Ossella C., Costici P.F. (2021). COVID-19 Changed the Incidence and the Pattern of Pediatric Traumas: A Single-Centre Study in a Pediatric Emergency Department. Int. J. Environ. Res. Public Health.

[25] Verdoni F., Ricci M., Di Grigoli C., Rossi N., Lombardo M.D.M., Curci D., Accetta R., Viganò M., Peretti G.M., Mangiavini L. (2021). Effect of the COVID-19 Outbreak on Pediatric Patients’ Admissions to the Emergency Department in an Italian Orthopedic Trauma Hub. Children.

[26] Trisolino G., Toniolo R.M., Marengo L., Dibello D., Guida P., Panuccio E., Evangelista A., Stallone S., Sansò M.L., Amati C. (2021). Resilience Against COVID-19: How Italy Faced the Pandemic in Pediatric Orthopedics and Traumatology. Children.

[27] Brayda-Bruno M., Giorgino R., Gallazzi E., Morelli I., Manfroni F., Briguglio M., Accetta R., Mangiavini L., Peretti G.M. (2021). How SARS-CoV-2 Pandemic Changed Traumatology and Hospital Setting: An Analysis of 498 Fractured Patients. J. Clin. Med..

[28] Agrawal S., Makuch S., Dróżdż M., Strzelec B., Sobieszczańska M., Mazur G. (2021). The Impact of the COVID-19 Emergency on Life Activities and Delivery of Healthcare Services in the Elderly Population. J. Clin. Med..

[29] Pokryszko-Dragan A., Chojdak-Łukasiewicz J., Gruszka E., Pawłowski M., Pawłowski T., Rudkowska-Mytych A., Rymaszewska J., Budrewicz S. (2021). Burden of COVID-19 Pandemic Perceived by Polish Patients with Multiple Sclerosis. J. Clin. Med..

[30] Polizzi C., Burgio S., Lavanco G., Alesi M. (2021). Parental Distress and Perception of Children’s Executive Functioning after the First COVID-19 Lockdown in Italy. J. Clin. Med..

[31] Frade F., Jacobsohn L., Gómez-Salgado J., Martins R., Allande-Cussó R., Ruiz-Frutos C., Frade J. (2021). Impact on the Mental and Physical Health of the Portuguese Population during the COVID-19 Confinement. J. Clin. Med..

[32] Lucki M., Wareńczak A., Chlebuś E., Daroszewski P., Lisiński P. (2021). The ICF Classification as a Simple Tool to Aid in the Assessment of Healthcare Services in a Non-COVID-19 Hospital during the COVID-19 Pandemic. Healthcare.

[33] Polan C., Burggraf M., Kauther M.D., Meyer H.-L., Rademacher F., Braitsch H., Jöckel K.-H., Hardes J., Streitbürger A., Dudda M. (2021). Development of Case Numbers during the COVID-19 Pandemic in a Center of Maximum-Care for Traumatology and Orthopedic Oncology. Healthcare.

[34] Placella G., Salvato D., Delmastro E., Bettinelli G., Salini V. (2020). CoViD-19 and ortho and trauma surgery: The Italian experience. Injury.

[35] Ciatti C., Maniscalco P., Quattrini F., Gattoni S., Magro A., Capelli P., Banchini F., Fiazza C., Pavone V., Pagliarello C.P. (2021). The epidemiology of proximal femur fractures during COVID-19 emergency in Italy: A multicentric study. Acta Biomed..

[36] Simon S., Frank B.J.H., Aichmair A., Manolopoulos P.P., Dominkus M., Schernhammer E.S., Hofstaetter J.G. (2021). Impact of the 1st and 2nd Wave of the COVID-19 Pandemic on Primary or Revision Total Hip and Knee Arthroplasty—A Cross-Sectional Single Center Study. J. Clin. Med..

[37] Czubak-Wrzosek M., Czubak J., Grzelecki D., Tyrakowski M. (2021). The Effect of the COVID-19 Pandemic on Total Hip and Knee Arthroplasty Surgical Volume in 2020 in Poland. Int. J. Environ. Res. Public Health.

[38] Kazubski K., Tomczyk Ł., Kopczyński B., Morasiewicz P. (2021). The Epidemiology of Hip and Knee Primary and Revision Arthroplasties during the COVID-19 Pandemic. Healthcare.

[39] Ulivi M., Orlandini L., Meroni V., D’Errico M., Fontana A., Viganò M., Mangiavini L., D’Anchise R., Parente F., Pozzoni R. (2021). Remote Management of Patients after Total Joint Arthroplasty via a Web-Based Registry during the COVID-19 Pandemic. Healthcare.

[40] Wainwright T.W. (2021). Enhanced Recovery after Surgery (ERAS) for Hip and Knee Replacement—Why and How It Should Be Implemented Following the COVID-19 Pandemic. Medicina.

[41] Giorgi P.D., Villa F., Gallazzi E., Debernardi A., Schirò G.R., Crisà F.M., Talamonti G., D’Aliberti G. (2020). The management of emergency spinal surgery during the COVID-19 pandemic in Italy. Bone Jt. J..

[42] Nuñez J.H., Sallent A., Lakhani K., Guerra-Farfan E., Vidal N., Ekhtiari S., Minguell J. (2020). Impact of the COVID-19 pandemic on an emergency traumatology service: Experience at a tertiary Trauma Centre in Spain. Injury.

[43] Schwartz J., King C.-C., Yen M.-Y. (2020). Protecting healthcare workers during the coronavirus disease 2019 (COVID-19) outbreak: Lessons from Taiwan’s severe acute respiratory syndrome response. Clin. Infect. Dis..

[44] De Georgeo M.R., De Georgeo J.M., Egan T.M., Klee K.P., Schwemm M.S., Bye-Kollbaum H., Kinser A.J. (2021). Containing SARS-CoV-2 in hospitals facing finite PPE, limited testing, and physical space variability: Navigating resource constrained enhanced traffic control bundling. J. Microbiol. Immunol. Infect..

[45] Yen M.-Y., Schwartz J., King C.-C., Lee C.-M., Hsueh P.-R. (2020). Society of Taiwan Long-term Care Infection Prevention and Control. Recommendations for protecting against and mitigating the COVID-19 pandemic in long-term care facilities. J. Microbiol. Immunol. Infect..

[46] Gandhi M., Yokoe D.S., Havlir D.V. (2020). Asymptomatic transmission, the Achilles’ heel of current strategies to control Covid-19. N. Eng. J. Med..

[47] Arons M.M., Hatfield K.M., Reddy S.C., Kimball A., James A., Jacobs J.R., Taylor J., Spicer K., Bardossy A.C., Oakley L.P. (2020). Presymptomatic SARS-CoV-2 infections and transmission in a skilled nursing facility. N. Eng. J. Med..

[48] Shih C.-L., Huang P.-J., Huang H.-T., Chen C.-H., Lee T.-C., Hsu C.-H. (2021). Impact of the COVID-19 pandemic and its related psychological effect on orthopedic surgeries conducted in different types of hospitals in Taiwan. J. Orthop. Surg..

[49] Lei S., Jiang F., Su W., Chen C., Chen J., Mei W., Zhan L.-Y., Jia Y., Zhang L., Liu D. (2020). Clinical characteristics and outcomes of patients undergoing surgeries during the incubation period of COVID-19 infection. EClinicalmedicine.

